# Mitochondrial transfer of mesenchymal stem cells effectively protects corneal epithelial cells from mitochondrial damage

**DOI:** 10.1038/cddis.2016.358

**Published:** 2016-11-10

**Authors:** Dan Jiang, Fei Gao, Yuelin Zhang, David Sai Hung Wong, Qing Li, Hung-fat Tse, Goufeng Xu, Zhendong Yu, Qizhou Lian

**Affiliations:** 1 Department of Ophthalmology, Li Ka Shing Faculty of Medicine, The University of Hong Kong, Hong Kong, China; 2 Department of Medicine, The University of Hong Kong, Hong Kong, China; 3 Shenzhen Institutes of Research and Innovation, University of Hong Kong, Hong Kong, China; 4 National Engineering Laboratory for Regenerative Medical Implantable Devices, 12 Yuyan Road, Guangzhou, China; 5 Central Laboratory, Peking University Shenzhen Hospital, Shenzhen, Guangdong, China

## Abstract

Recent studies have demonstrated that mesenchymal stem cells (MSCs) can donate mitochondria to airway epithelial cells and rescue mitochondrial damage in lung injury. We sought to determine whether MSCs could donate mitochondria and protect against oxidative stress-induced mitochondrial dysfunction in the cornea. Co-culturing of MSCs and corneal epithelial cells (CECs) indicated that the efficiency of mitochondrial transfer from MSCs to CECs was enhanced by Rotenone (Rot)-induced oxidative stress. The efficient mitochondrial transfer was associated with increased formation of tunneling nanotubes (TNTs) between MSCs and CECs, tubular connections that allowed direct intercellular communication. Separation of MSCs and CECs by a transwell culture system revealed no mitochiondrial transfer from MSCs to CECs and mitochondrial function was impaired when CECs were exposed to Rot challenge. CECs with or without mitochondrial transfer from MSCs displayed a distinct survival capacity and mitochondrial oxygen consumption rate. Mechanistically, increased filopodia outgrowth in CECs for TNT formation was associated with oxidative inflammation-activated NF*κ*B/TNF*α*ip2 signaling pathways that could be attenuated by reactive oxygen species scavenger *N*-acetylcysteine (NAC) treatment. Furthermore, MSCs grown on a decellularized porcine corneal scaffold were transplanted onto an alkali-injured eye in a rabbit model. Enhanced corneal wound healing was evident following healthy MSC scaffold transplantation. And transferred mitochondria was detected in corneal epithelium. In conclusion, mitochondrial transfer from MSCs provides novel protection for the cornea against oxidative stress-induced mitochondrial damage. This therapeutic strategy may prove relevant for a broad range of mitochondrial diseases.

Chemical burns to the cornea, including alkali injury, are a very common cause of severe corneal damage and vision impairment. Although more concentrated alkali injuries destroy all layers of the cornea, less concentrated alkali injuries also pose a threat to vision because oxidative stress- and inflammation-induced corneal mitochondrial damage often delays corneal repair. In the acute stage of a corneal chemical burn, management of anti-inflammatory and antiangiogenic factors and enhancing epithelial healing are critical to achieve a satisfactory clinical outcome. In cases with severe corneal damage, corneal stem cell transplantation is proposed to serve a novel strategy for corneal regeneration and scarring prevention. It has been documented that stem cell treatment accelerates regeneration of the corneal epithelial cells (CECs), restores the antioxidant protective mechanism and renews corneal optical properties.^1, 2, 3^ Increasing preclinical studies have indicated that transplantation of mesenchymal stem cells (or named multipotent stromal cells (MSCs)) offers several advantages in the repair of corneal damage.^1, 2^ First, MSCs are immune privileged, so there is no requirement for stringent human leukocyte antigen matching as in allogeneic transplantation.^4^ Second, MSCs can be acquired from many sources^5^ and can be prepared in various ‘off-the-shelf' formats for clinical application. Third, mitochondrial damage is often accompanied by increased inflammation. MSCs have the ability to migrate to an inflammatory site that makes them ideal candidates in targeted therapy.^6^ The mechanisms by which MSCs support corneal limbal stem cell growth and suppress alkali-induced oxidative injury in the cornea have been thought mainly to be via paracrine effects.^7, 8^ Some studies have indicated that cornea or bone marrow-derived MSCs can be induced to express markers of CECs.^1, 2, 9^ Nevertheless, apart from paracrine effects, anti-inflammation function and cell replacement, other mechanisms of MSC-modulated therapeutic effects in repair of ocular surface disease remain largely unknown.

Recently, our laboratory and others have discovered a novel mechanism of MSC-mediated airway epithelial repair directly through intercellular mitochondrial transfer.^6, 10^ We have also shown that human pluripotent stem cell-derived MSCs (iPSC-MSCs) have a significantly greater ability than bone marrow MSCs for mitochondrial donation to airway epithelial cells and greater efficiency in rescuing cigarette smoke-induced lung damage in rats.^6, 11^ We subsequently observed that efficient mitochndrial transfer is mainly associated with the formation of tunneling nanotubes (TNTs) that bridge MSCs and bronchoalveolar epithelial cells, forming tubular connections that allow direct intercellular communication.^6, 12^ Given the many similarities between cornea and airway epithelial cells, the question arises whether a similar mechanism is also employed in corneal repair following corneal injury. In this study, we aimed to determine whether MSC-mediated mitochondrial transfer, as in airway epithelail cells, could rescue the cornea from oxidative stress-induced mitochondrial damage. We established that MSCs can efficiently donate functional mitochondria and protect CECs from oxdative stress-induced damage through TNT formation. Furthermore, oxidative inflammation enhanced the efficiency of mitochondrial transfer from MSCs to stressed CECs and increased TNT formation that is regulated by the NF-*κ*B signaling pathway. Transplanation of MSCs scaffold onto a rabbit model of alkaline-induced corneal injury revealed that the efficacy of corneal repair is accompanied by MSC-mediated mitochondrial donation. The outcome of these proof-of-concept studies will enable us to develop a novel strategy to manipulate stem cell-based tissue repair by regulating direct mitochondrial transfer.

## Results

### Molecular tracking of intercellular mitochondrial transfer between MSCs and CECs

**Figure 1:**
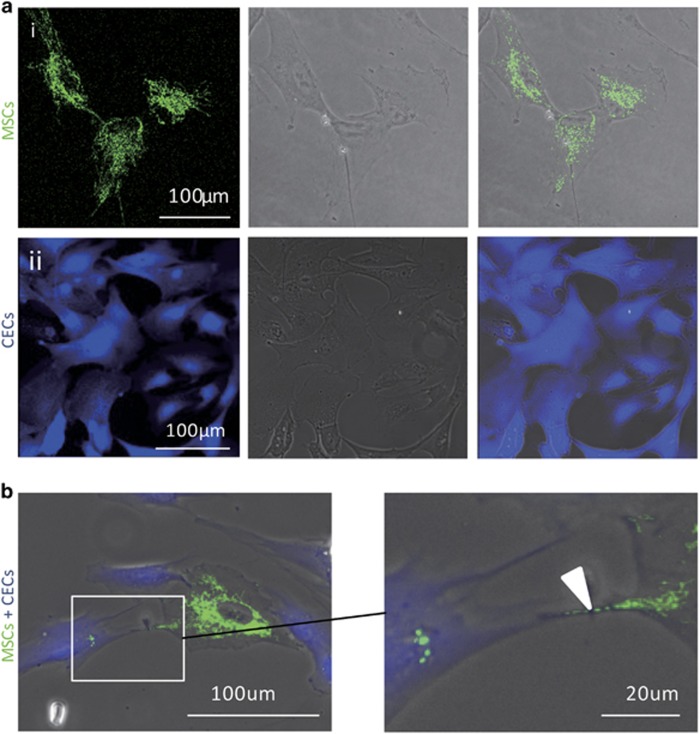
Intercellular mitochondrial transfer through TNTs during co-cultivation. (**a**) High yield of CECs and MSCs with molecular labeling. (i) MSCs labeled with Mito-GFP (green). (ii) CECs labeled with Violet. (**b**) Co-culturing of Violet-labeled CECs and mito-GFP-labeled MSCs for 24 h. Images show TNT formation and Mito-GFP-labeled mitochondria (arrow) were transferred from CECs to MSCs

To examine the potential of mitochondrial transfer from MSCs to CECs, we genetically labeled MSCs with lentiviral-mediated mitochondrial-specific fragment fused with green florescence protein (LV-Mito-GFP). LV-Mito-GFP vector encodes a mitochondrial cytochrome *c* oxidase subunit VIII and a fusion of GFP, allowing us to monitor mitochondrial trafficking (Figure 1a (i)). CECs were labeled with CellTrace Violet (Violet; Figure 1a (ii)). Under a fluorescent microscope, two distinct cell types could be observed, CECs (Violet) and iPSC-MSCs (green). LV-Mito-GFP-labeled MSCs and Violet-labeled CECs were co-cultured at a ratio of 1 : 1 for 24 h. After 24 h of co-culture, many Violet fluorescence-positive cells were co-located with Mito-GFP, indicating that mitochondria of MSCs had translocated to CECs (Figure 1b). Moreover, the Mito-GFP-positive organelles were transferred through the formation of TNTs between MSCs and CECs (Figure 1b).

### Enhanced effeciency of mitochondrial transfer from MSCs to CECs in Rotenone (Rot)-induced mitochondrial dysfunction of CECs

Recent studies have demonstrated that mitochondrial transfer from MSCs to the alveolar epithelium is a novel mechanism that protects against acute lung injury.^11^ We examined the potential of mitochondrial transfer from MSCs to CECs when CECs were exposed to Rot.^13^ Rot is a specific inhibitor of mitochondrial respiratory chain complex I and induces mitochondrial dysfunction by enhancing mitochondrial reactive oxygen species (ROS) production.^14, 15^ After 2 h of Rot pretreatment, CECs were seeded in XFe24 cell culture microplates (Agilent Technologies, Santa Clara, CA, USA) to examine mitochondrial oxygen consumption rate (OCR). Mitochondrial respiratory function of CECs was inhibited by Rot treatment in a dose-dependent manner, including basal, maximal and spare mitochondrial respiration as well as ATP production (Supplementary Figures S1A–E). Here a 0.5 *μ*M concentration of Rot treatment was sufficient to reduce 50% of mitochondrial respiratory function of CECs *in vitro* (Supplementary Figure S1A–E).

**Figure 2:**
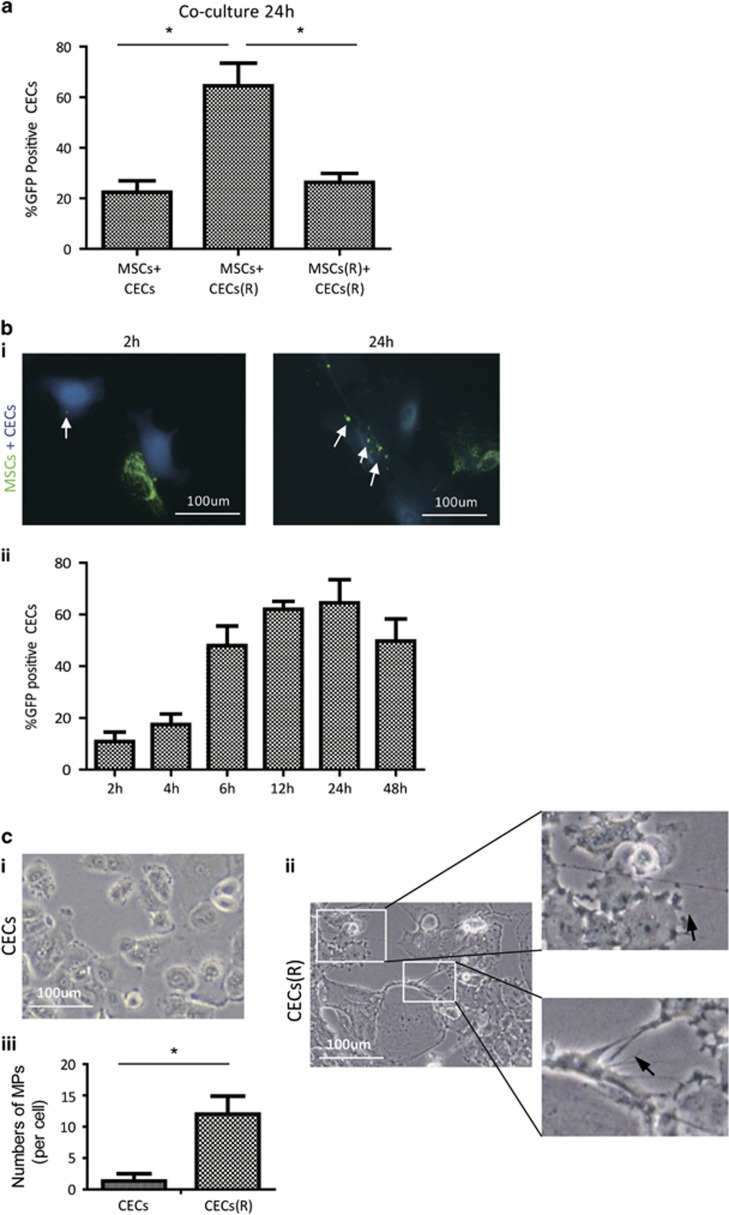
Enhanced efficiency of mitochondrial transfer from MSCs to CECs when CECs were exposed to Rot treatment. (**a**) Mitochondrial transfer ratio (Violet-positive cells containing mito-GFP/total Violet cells) was measured. Compared with CECs under normal conditions, Rot-pretreated CECs (CEC(R) ) enhanced mitochondria transfer of MSCs when co-cultured (MSCs+CECs *versus* MSCs+CECs(R), *P<0.05, *n=*100). The enhanced mitochondrial transfer was attenuated when MSCs were pretreated with Rot (MSCs+CECs(R) *versus* MSCs(R)+CECs(R), *P<0.05, *n=*100). (**b**) (i) Mito-GFP-labeled MSCs and Violet-labeled CECs were co-cultured at 1:1 ratio. Representive images of intercellular mitochondrial transfer between CEC(R) and MSCs at 2 and 24 h of co-culture. (ii) Mitochondrial transfer ratio was measured over time from 2, 4, 6, 12, 24 to 48 h during co-culture of CECs(R) and MSCs. (**c**) (i) Rare membrane protrusions (MPs) were observed when CECs were in normal conditions without Rot treatment. (ii) Representative images showing a lot of MPs when CECs were pretreated with Rot. (iii) Quantitative analsyis of MP formation between CECs and Rot pretreated CECs (CECs(R) *versus* CECs, *P<0.05)

Next we examined the mitochondrial transfer efficiency from MSCs to CECs under normal or Rot-treated conditions. Compared with CECs without Rot treatment, an enhanced mitochondrial uptake was observed when CECs were exposed to Rot treatment, suggesting that injured CECs had an increased uptake of mitochondria from MSCs (Figure 2a). We performed further quantitative assessment of the mitochondrial transfer ratio after 2, 4, 6, 12, 24 and 48 h. The fixed and stained samples were studied using laser scanning confocal microscope (LSM710; Carl Zeiss AG, Oberkochen, Germany). The mitochondria of MSCs could be transferred to CECs as early as 2 h after co-culture, reaching a peak at 24 h and declining at 48 h (Figures 2b (i and ii)). Furthermore, 25–50 nM of Rot treatment could reduce mitochondrial respiratory function in MSCs by 50% (Supplementary Figures S2A–E). When MSCs were treated with Rot, co-culture of these Rot-treated MSCs (MSCs(R)) with CECs did not show increased mitochondrial transfer from MSCs to CECs, suggesting that Rot-induced mitochondrial damage in CECs, rather than in MSCs, resulted in enhanced mitochondrial transfer from MSCs to CECs (Figure 2a). These results highlight that functional mitochondrial transfer can strongly protect CECs against Rot-induced mitochondrial damage, and those MSCs with impaired mitochondria (i.e., aged MSCs) may not be desirable as therapeutic donor cells. Moreover, we observed morphological changes to CECs when exposed to 0.5 *μ*M of Rot. The average number of membrane protrusions was increased in Rot-treated CECs compared with CECs under normal conditions (Figures 2c (i–iii)), suggesting that oxidative stress may be responsible for the formation of TNTs.

### MSC-donated mitochondria functionally protect CECs against Rot-induced mitochondrial damage

**Figure 3:**
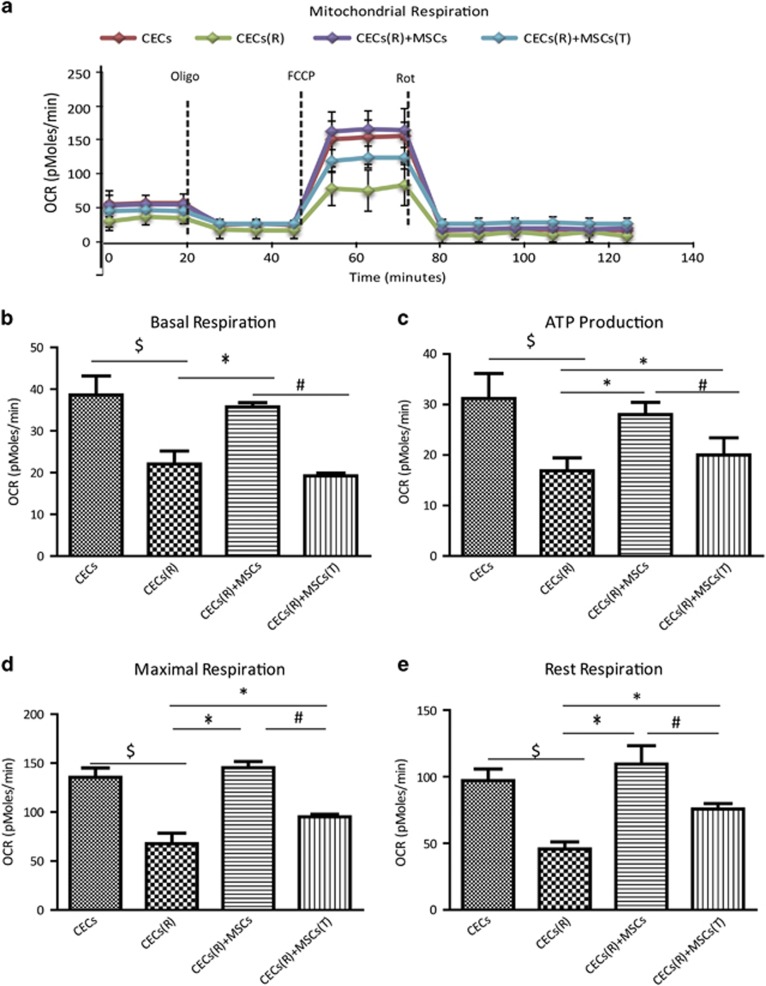
MSC-transferred mitochodria improve mitochondrial respiratory function of CECs. OCR of sorted CECs, CECs(R), CECs(R) co-cultured with MSCs (CECs(R)+MSCs) and CECs(R) co-cultured with MSCs in Transwell (CECs(R)+MSCs(T)) for 24 h were measured over time (mins) by extracellular flux analyzer. (**a**) Fifteen total OCR measurements were taken over 2 h: 3 basal respiration, 3 Oligomycin-sensitive respiration, 3 maximal respiratory capacity, and 6 non-mitochondrial respiration. The *x* axis in panel (**a**) describes the measurement (mins). (**b**) Basal mitochondral OCR of CECs from different groups. (**c**) ATP production of CECs from different groups. (**d**) Maximal respiration of CECs from different groups. (**e**) Rest respiration of CECs from different groups (^$^*P*<0.05 *versus* CECs; *P<0.05, *versus* CECs(R); ^#^*P*<0.05 *versus* CECs(R)+MSCs(T))

To determine whether mitochondria transferred from MSCs to CECs are functional, we compared mitochondrial respiratory function by measuring the cellular OCR of mitochondrial-transferred and non-mitochondrial-transferred CECs after Rot challenge. CECs were labeled with Violet and MSCs with LV-Mito-GFP. CECs and MSCs were co-cultured at a ratio of 1:1 for 24 h. Violet-labeled and LV-Mito-GFP-positive CECs were separated by cell sorting. The separated CECs with Violet and GFP double positive were taken as mitochondrial-transferred CECs. These CECs were seeded in XFe24 cell culture microplates to examine OCR using an extracellular flux analyzer. Compared with CECs under normal conditions, basal mitochondrial respiration, ATP production, rest respiration and maximum respiration were significantly decreased in all of the Rot-treated CECs (CECs(R)) (Figures 3b–e). In contrast, respiratory function were markedly improved in those CECs with mitochondria transferred from MSC co-cultivation (Figures 3a–e). Moreover, healthy mitochondrial respiratory function is the cornerstone of cellular bioenergetic homeostasis, having leading roles in cell apoptosis, proliferation and a number of biosynthetic pathways. MTT assays show that MSC-mediated mitochondrial transfer protects CECs from Rot-induced cell death and proliferation inhibition (Supplementary Figure S4).

It is well documented that paracrine factors of MSCs largely contribute to tissue repair.^16, 17, 18^ Next we questioned whether the mitochondrial transfer manner of MSCs protects CECs and is different from paracrine action. We seeded Rot-treated CECs in the upper chamber of a Transwell unit, with MSCs seeded in the lower chamber to provide paracrine factors. After 24 h of co-culture, CECs were harvested and subjected to OCR analysis. Compared with mitochondrial-transferred CECs, CECs harvested from Transwell units displayed much less bioenergetics preservation (Figures 3a–e), indicating that mitochondrial transfer from MSCs truly protected CECs against Rot insults. In addition, CECs harvested from Transwell co-culture unit displayed improved OCR parameters compared with Rot-treated CECs (CECs(R)), suggesting that the paracrine effects of MSCs also partially protect CECs against Rot-induced mitochondrial dysfunction.

### Rot-induced ROS activates NF-*κ*B in CECs and enhances TNTs formation via upregulation of TNF*α*ip2

**Figure 4:**
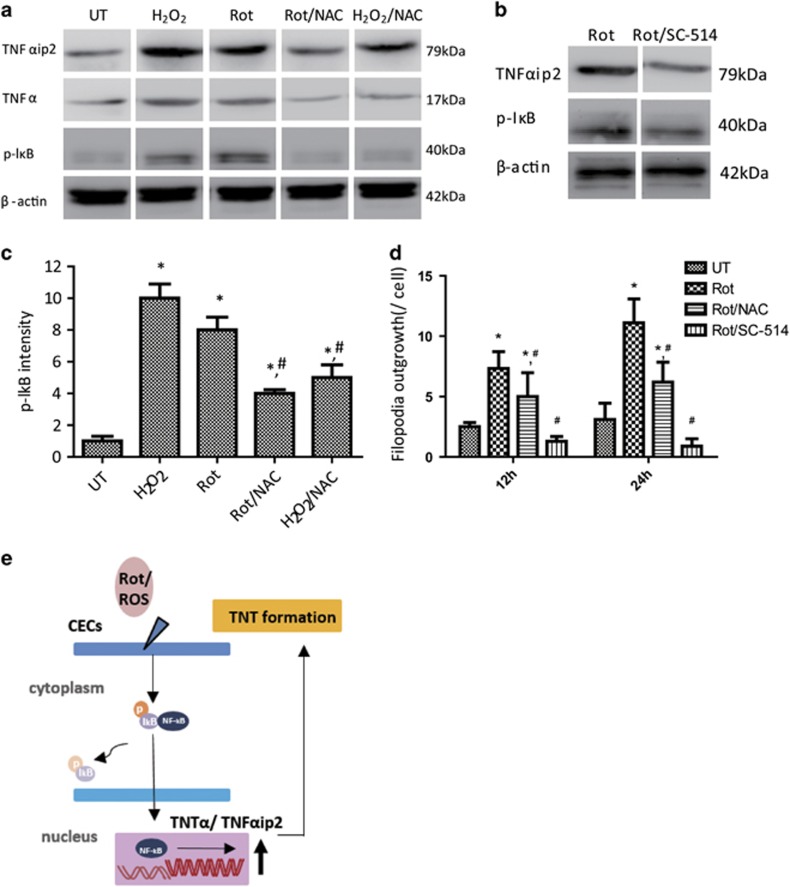
Rot-induced ROS activates NF-*κ*B in CECs and enhances TNT formation via upregulation of TNF*α*ip2. (**a**) Western blotting showing the level of p-I*κ*B, TNF*α* and TNF*α*ip2 in different groups. (**b**) p-I*κ*B and TNF*α*ip2 expression induced by Rot was strikingly decreased in hCECs with SC-514. (**c**) Quantification of p-I*k*B intensity. The levels of phosphorylation were normalized to *β*-Actin. Results were obtained from three independent experiments (*P<0.001 *versus* UT; ^#^*P*<0.05 *versus* Rot). (**d**) hCECs were treated with Rot, Rot plus NAC or Rot plus SC-514 for 12 and 24 h; the number of Filiopodia outgrowth per cell was calculated (*P<0.001 *versus* UT; ^#^*P*<0.05 *versus* Rot, *n=*100). (**e**) The proposed mechanism whereby Rot-induced ROS activates NF-*κ*B and enhances TNT formation via upregulation of TNF*α*ip2 in hCECs

It has been demonstrated that TNF*α*ip2 can stimulate the formation of TNTs.^14, 19^ We attempted to determine whether Rot-induced ROS could activate NF-*κ*B and enhance TNT formation via upregulation of TNF*α*ip2 in primary human CECs (hCECs). In agreement with previous reports, when hCECs were treated with Rot or hydogen peroxide (H_2_O_2_), the phosphorylated level of NF-*κ*B subunit p-I*κ*B, TNF*α* and TNF*α*ip2 was markedly enhanced (Figures 4a and c) accompanied by a significant increase in TNTs per cell as measured by filopodia outgrowth (Figure 4d). In contrast, when hCECs were pretreated with ROS scavenger *N*-acetylcysteine (NAC; 5 mM) for 1 h following Rot or H_2_O_2_ treatment, a largely decreased level of p-I*κ*B, TNF*α* and TNF*α*ip2 was evident compared with hCECs treated with H_2_O_2_ or Rot only (Figure 4a). The reduced phosphorylation state of p-I*κ*B was accompanied by decreased TNT formation (Figures 4c and d), suggesting that Rot-induced ROS activates NF-*κ*B and enhances TNT formation by upregulation of TNF*α*ip2 in hCECs (Figure 4d).

To further confirm whether Rot-induced TNF*α*ip2 for TNT formation is mediated through NF-*κ*B pathway, the phosphorylated level of NF-*κ*B subunit p-I*κ*B and TNT formation in hCECs were examined under the treatment of NF-*κ*B inhibitor SC-514. Rot treatment promoted TNT formation (Figure 4d), while the level of p-I*κ*B and TNF*α*ip2 was also significantly increased (Figure 4b). In contrast, NF-*κ*B inhibitor SC-514 (1 *μ*M) treatment significantly attenuated the level of p-I*κ*B and TNF*α*ip2 and abolished TNF*α*ip2-induced TNT formation (Figure 4d), suggesting that Rot/NF-*κ*B/TNF*α*ip2 signaling pathway is predominantly involved in TNF*α*ip2-mediated TNT formation of hCECs.

### Evidence of mitochondrial transfer from MSCs following transplantation of MSC scaffold in corneal repair

**Figure 5:**
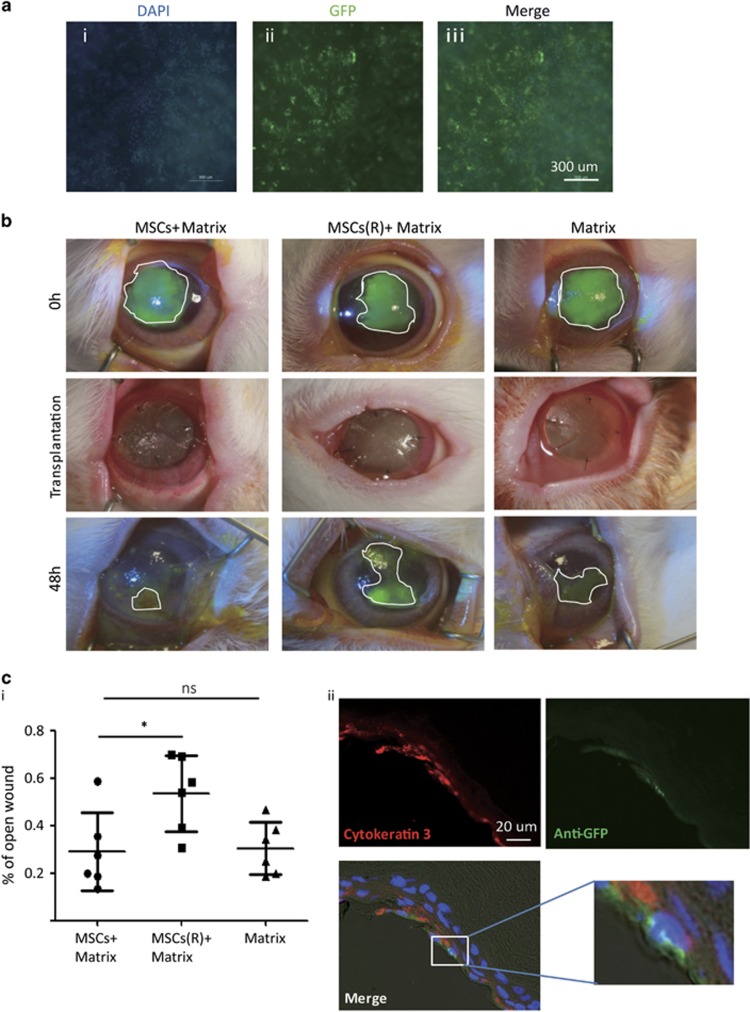
MSC transplanation and mitochondrial transfer *in vivo*. (**a**) Images of MSC culture on acellular porcine cornea matrix. After 72 h culture, the inside surface of matrix was covered by MSCs. Representative images of (i) DAPI (4,6-diamidino-2-phenylindole), (ii) GFP and (iii) Merge. (**b**) Slit-lamp images of MSCs on Matrix (MSC Matrix), on Rot-pretreated MSC Matrix (MSC(R)+Matrix) and Matrix only group cornea. Transplantation of the above matrix was performed following Alkali-induced corneal injuries. Matrix was removed and corneal epithelial fluorescein staining was performed at 48 h after transplantation. (**c**) (i) The statistical data of the rest of corneal erosion after 48 h matrix transfer; mitochondria donation from MSCs promoted corneal epithelium healing but the healing effort significantly declined if MSCs were pretreated with Rot. (ii) Representative images of immunofluorescent staining of anti-GFP (green) and CK3 (red) performed on the corneal tissue of MSC Matrix groups. Mitochondria from MSCs (green) were detected within CECs (amplified). (*P<0.05; NS, not significant)

To examine whether mitochondrial transfer of MSCs occurs *in vivo* and contributes to tissue repair, we cultured Mito-GFP-labeled MSCs either under normal or Rot-treated conditions on the surface of decellularized porcine cornea matrix (Figures 5a (i–iii); Supplementary Figures S3A and B). After 72 h of MSC culture, the matrix covered with MSCs was transplanted onto injured rabbit cornea.

As shown in a previous study,^20^ corneal mitochondrial dysfunction was induced by alkaline burn.^13^ Briefly, 0.5 mol sodium hydroxide solution-soaked sized filter papers were applied to inflict a 8-mm diameter alkali burn to the center of the cornea (Supplementary Figure S3C and Figure 5b, 0h). Three groups of engineered matrix were compared for transplantation to the center of the cornea after alkali exposure and irrigation (Figure 5b, transplantation): (1) MSCs+matrix, (2) Rot-treated MSCs matrix group, MSCs(R)+matrix, and (3) matrix. Forty-eight hours after surgery, recovery of the corneal epithelium was assessed by corneal epithelial fluorescein staining (Figure 5b, 48 h). Among the three groups, the MSCs+matrix group achieved the best wound healing compared with the MSC(R)+matrix group (*P<0.05) or matrix only group (*P*=0.06) (Figure 5c (i)). The results show that transplantation with MSCs+matrix, but not with Rot-treated MSCs matrix, improved corneal wound healing, suggesting that only healthy MSCs provides beneficial effects for corneal wound recovery. In addition, compared with Matrix transplantation, MSCs+matrix transplantation only showed further marginally better effects but not yet significant differences in statistics for corneal wound healing (*P*=0.06, Student's *t*-tests). This suggests that the healing effort of MSCs significantly declined if mitochondrial function of MSCs was impaired.

To determine whether there was mitochondrial transfer from MSCs to rabbit cornea following transplantation, eyes were isolated, fixed and sectioned for histological examination at 48 h after transplantation. This short time of transplantation is unlikely to induce differentiation of MSCs to CECs. Immunostaining of Cytokeratin 3 (CK3) and anti-GFP was used to identify CECs and Mito-GFP-labeled mitochondria, respectively (Figure 5c (ii)). Hoechst was used for nuclei counterstain. It revealed that some GFP-positive mitochondria were detected within CK3-positive CECs only, excluding contamination from MSCs. It indicated that MSCs could transfer mitochondria to CECs *in vivo*.

## Discussion

The failure of appropriate corneal repair following severe injury, such as chemical burn injuries, often leads to loss of vision. MSCs have been proposed as a valuable cell source to prevent corneal scar formation and promote corneal wound healing.^17, 21^ MSCs exert their therapeutic function mainly through paracrine, anti-inflammatory actions and corneal cell differentition or cell replacement.^1, 2, 3, 22^ Several studies have demonstrated that mitochondrial transfer is an important mechanism of MSC therapy.^6, 14, 23^ In this study, we provide first *in vitro* and *in vivo* evidence that MSCs can exert their corneal protection function via efficient functional mitochondrial donation. These results also reveal an enhanced efficiency of mitochondrial transfer from MSCs to CECs when CECs are under stressful conditions. Indeed, inhibition of NF-*κ*B pathway by inhibitor SC-514 (1 *μ*M) reduces the efficiency of TNT formation, suggesting that Rot/NF-*κ*B/TNF*α*ip2 signaling pathway is predominantly involved in TNF*α*ip2-mediated TNT formation.

The mechanisms by which MSCs support corneal tissue regeneration have been thought mainly to be via paracrine effects.^7, 8, 24^ Our study indicates that functional mitochondrial transfer from MSCs which directly protects CECs against Rot-induced mitochondrial damage is another important mechanism. In addition, when MSCs were co-cultured with CECs in Transwell units, non-mitochondrial-transferred CECs also exhibited strong resistance to Rot-induced damage, suggesting the paracrine action of MSCs is also critical in corneal protection. Nevertheless, the crosstalk between paracrine action and mitochondrial transfer is an interaction of two independent processes with consequent MSC-mediated CEC protection.

In addition, our study demonstrates that Rot treatment can increase TNT formation and enhance intercellular mitochondria transfer between MSCs and CECs, in which the NF-*κ*B signaling pathway is predominantly involved in TNT development for MSC-mediated mitochondrial transfer. Indeed, NF-*κ*B activation increased TNF*α*ip2 and triggered F-actin polymerization that may subsequently upregulate TNT formation through actin-driven protrusions of the cytoplasmic membrane in MSCs.^19, 25^ This, in turn, contributes to mitochondrial donation from MSCs to CECs.

We also observed that mitochondria transfer of MSC to CECs *in vivo* and only healthy MSC matrix, but not mitochondrial dysfunctional MSC, provides beneficial effects for corneal wound recovery, suggesting that the quality of MSCs is very critical for tissue repair. In the acute stage of a corneal chemical burn, enhancing epithelial healing and management of anti-inflammatory are critical to achieve a satisfactory long-term clinical outcome, such as prevention of corneal scarring.

There are several limitations in this study. First, compared with Matrix transplantation, MSC matrix transplantation only showed further marginally better effects but not yet significant differences for corneal wound healing (*P*=0.06, Student's *t*-tests). The weakness may be attributed to a limited number size (*n=*6) or MSCs lost on matrix after transplantation onto corneal surface because only a few mitochondria of MSC can be detected after 48 h of transplantation. In future study, more suitable matrix as the carrier to deliver MSCs should be optimized (i.e., contact lens and amniotic membrane).

Second, although our data demonstrated an obligatory role of NF-*κ*B signaling in regulating mitochondrial transfer via TNT formation, other mechanisms that facilitate intercellular mitochondrial movement are being investigated. Recently, increasing reports indicate many ways that mitochondria can be donated by MSCs to recipient cells via MSCs to macrophages,^26^ via a formation of a gap junction^11^ or via microvesicle secretion.^27^ More signaling pathways involved in intercellular mitochondrial donation deserve further investigation.

In summary, our study reveals the protective effects of MSCs against corneal injury via efficacious mitochondrial transfer from MSCs to CECs, thus augmenting alkaline burn-induced corneal damage. The outcome of this study opens a novel view of stem cell-based treatment on ophthalmology disease that may help develop a novel strategy to manipulate direct mitochondrial transfer of stem cells for ocular surface repair.

## Materials and Methods

### Primary cell culture and culture condition

Human iPSC-MSCs were prepared and cultured according to the protocol previously described.^28^ In brief, cells were cultured in Knockout Dulbecco modified Eagle's medium (DMEM; Gibco, Invitrogen, Carlsbad, CA, USA), 15% fetal bovine serum (FBS), 2 ng/ml EGF and 2 ng/ml FGF.^28^ Primary hCECs (C018-5c, Life Technologies, Gaithersburg, MD, USA) were cultured according to the published protocol.^29^ hCECs were maintained at 37 °C, 5% CO_2_, in Keratinocyte–SFM medium (Cat. No.:17005-042, Gibco) and were used between passages 2–5. The rabbit CECs were prepared according to the published protocol^6^ with some modifications. The corneas of the non-experimental whole New Zealand rabbit were harvested and washed twice with Hank's balanced salt solution buffer. The tissue was cut into 2 × 2 mm^2^ small pieces after removal of excessive corneal stroma and placed onto a six-well culture dish and Keratinocyte–SFM medium. The cultured CECs were identified by the expression of CK3.

### CEC oxidative stress model and *in vitro* co-culture system

To examine the inhibition of mitochondrial respiratory function, CECs were treated with 0.05, 0.1, 0.5 and 1 *μ*M concentration of Rot for 2 h. Selection of Rot concentration was determined by OCR measurement. CECs with or without Rot treatment were co-cultured with MSCs at 1:1 ratio. The mixed cells were seeded at a density of 1 × 10^4^/cm^2^ and supplemented with 1:1 MSC medium (DMEM with 15% FBS, 2 ng/ml EGF, 2 ng/ml FGF) and CEC medium (Keratinocyte–SFM).

### Immunocytochemistry and immunofluorescence

For live cell imaging, MSCs were transfected with LV-Mito-GFP. CECs were labeled with Violet (Cat No. C34557) before co-culture for a time-lapse video assay or fluorescence-activated cell sorting. After fixation, cells or tissue section slides were fixed and processed as previously described.^30^ After permeabilization with 1% Triton X-100 for 15 min, samples were incubated with 5% bovine serum albumin for 30 min. Then cells or tissue sections were stained with primary antibodies and incubated overnight at 4 °C in a 1:100 dilution. Negative control reactions were incubated with phosphate-buffered saline instead of the primary antibody. The second antibody anti-mouse IgG, anti-rabbit IgG or anti-goat IgG (1:1000) was then used against the primary antibodies. The primary antibodies used in this study were monoclonal mouse anti-CK3 antibody (SC-80000, Santa Cruz Biotechnology Inc., Dallas, TX, USA) and anti-GFP (sc-8334, Santa Cruz Biotechnology, Inc.,).

### Assessment of mitochondrial transfer

Mitochondrial targeting green fluorescence protein (Mito-GFP, Cat. No.: Cyto102-PA-1, System Biosciences, Palo Alto, CA, USA) was transfected according to the protocol. Briefly, 2 × 10^5^ iPSC-MSCs were seeded 1 day before infection. The following day, LV-Mito-GFP was added to 1 ml growth medium and incubated at 37 °C with a constant supply of 5% CO_2_ for 16 h. CECs were labeled with Violet. The mitochondrial transfer was examined using laser scanning confocal microscope LSM710 or a time-lapse video recorder.

Quantitative analysis of the mitochondrial transfer ratio from MSCs to CECs was analyzed by counting double-positive cells among 100 CECs (*n=*5). Briefly, Violet-labeled CECs and Mito-GFP-labeled MSCs were co-cultured at a ratio of 1:1 in a 24-well plate with or without challenge of Rot and examined after 2, 4, 6, 12, 24 and 48 h. Data were acquired by laser scanning confocal microscope LSM710 with a 488 nm argon laser and a 405 nm laser and analyzed using the Image J software (version 1.48v, Wayen Rasband, National Institutes of Health, Bethesda, MD, USA; http://imagej.nih.gov/ij).

### Transwell assay

Transwell assay was performed to determine the role of mitochondrial transfer in improvement of mitochondrial respiratory function of CECs and whether this mitochondrial transfer-mediated improvement is independent of the paracrine factors secreted by MSCs. CECs (1 × 10^5^ per well) were seeded on the lower chamber and MSCs (1 × 10^5^ per well) were seeded on the upper chamber. CECs in Transwells were harvested for measurement of OCR following different treatments: (1) CECs treated with Rot and co-cultured with MSCs for 24 h (CECs(R)+MSCs group); (2) CECs treated with Rot and co-cultured with MSCs in Transwell (CECs(R)+MSC(T) group); (3) CECs treated with Rot (CECs(R)) or (4) intact CECs group. Cell sorting was performed after 24 h of co-culture followed by OCR measurement.

### OCR measurement

Bioenergetic analysis of OCR, an indicator of mitochondrial respiration, of CECs was measured using the Seahorse XFe24 extracellular flux analyzer (Agilent Technologies, Santa Clara, CA, USA) as described previously.^31, 32^ The bioenergetic profile comprised of four measurements: (i) basal mitochondrial OCR was measured in medium containing 5.78 mM D-glucose, 0.6 mM Pyruvate and 6.98 mM L-glutamin; (ii) after inhibition of ATP synthase using oligomycin (1 *μ*M), ATP synthesis turnover and respiration-driving proton leak were assessed by measuring OCR; (iii) after treatment with the uncoupling agent carbonyl cyanide-p-trifluoromethoxyphenylhydrazone (2 *μ*M), maximal mitochondrial respiratory capacity was determined by measuring OCR; and (iv) finally, non-mitochondrial respiration was assessed by measuring OCR after treatment with complex I inhibitor: Rot (2 *μ*M).

### Western blotting

Western blotting for TNF*α*ip2, TNF-*α*, *β*-Actin and p-I*κ*B was performed as previously described.^33^ Briefly, the concentration was measured with the Bradford method (Bio-Rad Protein Assay kit; Bio-Rad Laboratories, Hercules, CA, USA) and then run on 10% polyacrylamide gel. Before protein transfer, PVDF membranes were incubated with the primary antibodies overnight at 4 °C and then incubated with horseradish peroxide-conjugated anti-rabbit or anti-mouse secondary antibody at 37 °C for 1 h. Primary antibodies were: TNF*α*ip2 (SC-28318, Santa Cruz Biotechnology, Inc.), p-I*κ*B (sc-8404, Santa Cruz Biotechnology, Inc.), TNF *α* (ab6671, Abcam, Cambridge, MA, USA) and anti-*β*-Actin (ab8227, Abcam).

### Preparation of MSC scaffolds

Acellular porcine cornea matrix was used as a scaffold for MSC culture (kind gifts from Grandhope Biotechnolohy Limited, Guangzhou, China). After washing with sterile PBS, scaffolds were incubated in basal culture media for 24 h. MSCs in passage 8 were plated onto the top of scaffold in 24-well plates. Matrix was divided into three groups, MSCs group (MSCs Matrix), MSCs pretreated with Rot (50 nmol) group (MSCs(R) Matrix) and acellular group (Matrix). The density of seeded cells was 1 × 10^5^ cells per scaffold. Seeded MSCs were cultured for 72 h in MSC medium for futher transplantation experiment.

### Animals models and MSC scaffold transplantation

This study was conducted in accordance with the Guidelines for Animal Experiments and approved by the University Animal Ethics Committee of the University of Hong Kong and the ARVO Statement for the Use of Animals in Ophthalmic and Vision Research. A corneal alkali burn was generated in the right eye of each rabbit as discribed previously.^13^ A rabbit model of corneal alkaline burn was induced in adult rabbits (3–4 months) by appling a piece of filter paper (8-mm diameter) soaked with NaOH (0.5 mol/l) to the center of the cornea for 40 s after the rabbits were anesthetized. Then the cornea was rinsed with 50 ml of physiological saline for 1 min immediately. Subsequently, the rabbits were randomly divided into three groups: group 1, alkaline burn and MSC matrix transplantation (MSCs+Matrix, *n=*6); group 2, alkaline burn and Rot-pretreated MSC matrix transplantation (MSCs(R)+Matrix, *n=*6); and group 3, alkaline burn and matrix transplantation (Matrix, *n=*6). The left eye served as a control. After irrigation, MSCs+matrix, MSCs(R)+matrix or matrix were transplanted onto the corneal surface to completely cover the burned area and sutured with 10-0 nylon. The rabbits were killed 48 h after transplantation.

### Corneal repairs and tissue analysis

On days 0 and 2, the eyes of each group were examined using slit lamp. To measure the corneal defect, following topical anesthesia with Tetracaine Hydrochloride Ophthalmic Solution 0.5% (Alcon, Puurs, Belgium) and a fluorescein dye strip (Negah Fluorescein Paper; Jingming Co., Tianjin, China) was placed in the inferior fornix. A photograph was taken of the corneal epithelial defect using a 18.7 megapixel high-resolution digital camera (600D; Canon, Tokyo, Japan) at × 4 optical zoom. After corneal examination, rabbits were killed with intravenous pentobarbital sodium (100 mg/kg, Alfasan International B.V., Woerden, Holland) on day 2. Eyes were isolated and fixed in 4% PFA and sent for histopathology. After tissue processing and embedding in paraffin blocks, 5-*μ*m tissue sections were prepared and stocked for futher analysis.

### Statistical analysis

Statistical analysis was performed using the Prism 5.04 Software (GraphPad Software for Windows, San Diego, CA, USA), and results are reported as mean±S.D. Comparisons between more than two groups were analyzed by one-way ANOVA test. Comparisons between two groups were performed using Student's *t*-test. *P*-value<0.05 was considered significant.

## Abbreviations

CEC: corneal epithelial cell
CK3: Cytokeratin 3
hCEC: human corneal epithelial cell
H_2_O_2_: hydogen peroxide
iPSC-MSC: pluripotent stem cell-derived MSC
LV-Mito-GFP: lentiviral-mediated mitochondrial-specific fragment fused with green florescence protein
MSC: mesenchymal stem cell
NAC: *N*-acetylcysteine
OCR: oxygen consumption rate
ROS: reactive oxygen species
Rot: Rotenone
TNT: tunneling nanotube
Violet: CellTrace Violet
