# Supplementary material for: Mitochondrial transfer of mesenchymal stem cells effectively protects corneal epithelial cells from mitochondrial damage
Source: Cell Death Dis. 2016 Nov 10;7(11):e2467–. doi: 10.1038/cddis.2016.358 (PMC5260876; doi:10.1038/cddis.2016.358)
Supplement: Supplemental Data [file cddis2016358x1.docx]

Mitochondrial transfer of Mesenchymal Stem cells effectively protects corneal epithelial cells from mitochondrial damage

Dan Jiang^1^, Fei Gao^1^, Yuelin Zhang^2^, David SH Wong^1^, Qing Li^1^, Hung-fat Tse^2,3^, Goufeng Xu^5*^, Zhendong Yu^4^*, Qizhou Lian^1, 2,3^*

^1^Department of Ophthalmology, Li Ka Shing Faculty of Medicine, the University of Hong Kong, Hong Kong;

^2^Department of Medicine, the University of Hong Kong, Hong Kong;

^3^ Shenzhen Institutes of Research and Innovation, University of Hong Kong, China;

^4^Central Laboratory, Peking University Shenzhen Hospital, Shenzhen, Guangdong, China;

^5^National Engineering Laboratory for Regenerative Medical Implantable Devices, 12 Yuyan Road，Guangzhou, China.

**Running Title:** Intercellular Crosstalk Improves Corneal Healing

***Address correspondence to:**

Qizhou Lian, MD, PhD.

Department of Ophthalmology, Li Ka Shing Faculty of Medicine

The University of Hong Kong, Hong Kong

Tel: +852-21899752 Fax: +852-28162095

Email: qzlian@hkucc.hku.hk (Q Lian);

or [dongboyaa@163.com(Z](mailto:dongboyaa@163.com(Z) Yu);

or xuguofeng@guanhaobio.com (G Xu)

**Supplemental Methods and Figure legends**

**Materials and Methods:**

**MTT assay**

Cell viability was measured with colorimetric determination of the conversion of  MTT (3-(4,5-Dimethylthiazol-2-yl)-2,5-diphenyltetrazolium bromide) assay (5 mg/ml; Sigma) into MTT formazan crystals by mitochondrial dehydrogenases of viable cells[[1](#_ENREF_1)]. Formazan crystals were dissolved in DMSO and measured at a wavelength of 490 nm.

Cells plated at densities of 1, 2, 3, 4, 5, 6, 7, 8, 9, 10, 20×10^3^ cells/100 μl per 0.3 cm^2^ and grown for 24 h indicated a linear relationship between absorbance and cell density (r^2^=0.9894).

**Legends:**

**S1 Impairment of mitochodrial respirational function in CECs exposed to rotenone.**

A) Oxygen consumption rate(OCR) of CECs under the treatment of different concentration of rotenone(Rot) was measured over time(min) by extracellular flux analyzer. B) Basal respiration of CECs under diffetent treatment was calculated. C) ATP production of CECs in different condition was calculated. D) Maximal respiration of CECs in different condition was calculated. E) Spare respiration of CECs in different condition was calculated.(*, p<0.05, **,p<0.01, ***,p<0.001 vs. Control.)

**S2 Impairment of mitochodrial respirational function in MSCs exposed to rotenone.**

A) Eleven total OCR measurements were taken over 3 h: 3 basal respiration before treatment, 15 basal respiration after 0nM, 25 nM, 50 nM and 100 nM, Rot injection, 3 Oligomycin-sensitive respiration, 3 maximal respiratory capacity, and 3 non-mitochondrial respiration. The x-axis in (A) describes the measurement min. Oxygen consumption rate(OCR) of CECs under the treatment of different concentration of rotenone(Rot) was measured over time(min) by extracellular flux analyzer. B) Basal respiration of CECs under diffetent treatment was calculated. C) ATP production of CECs in different condition was calculated. D) Maximal respiration of CECs in different condition was calculated. E) Spare respiration of CECs in different condition was calculated.(*p<0.05, **p<0.01, ***p<0.001 vs. Control.)

**S3** **Rabbit corneal alkaline burn model and cell scaffolds construction**

A) Acellular porcine cornea matrix with immuno blockades (9-10 mm diameter). B) MSCs plated onto the top of scaffolds in 24-well plates and cultured for 72h. C) Rabbit corneal alkaline burn model i) filter paper (8-mm diameter) ii) Normal rabbit corneal iii) filter paper soaked with NaOH (0.5 mol/l) on the center of the cornea for 40 seconds. iv) corneal central cloudy after alkaline burn.

**S4 Cell proliferation of CECs were measured by MTT assay.**

A) Standard curve of CECs using MTT Cell proliferation Assay. Linear relationship between absorbance and cell number (r^2^=0.9894)

B) The cell proliferation of post-sorting CECs measured in all of the treatment groups (CECs(R), CECs(R)+MSCs, CECs(R)+MSCs(T),) compared with the control group(CECs). (*, p<0.05)
