# Supplementary figures and images for: Mitochondrial transfer of mesenchymal stem cells effectively protects corneal epithelial cells from mitochondrial damage
Source: Cell Death Dis. 2016 Nov 10;7(11):e2467–. doi: 10.1038/cddis.2016.358 (PMC5260876; doi:10.1038/cddis.2016.358)

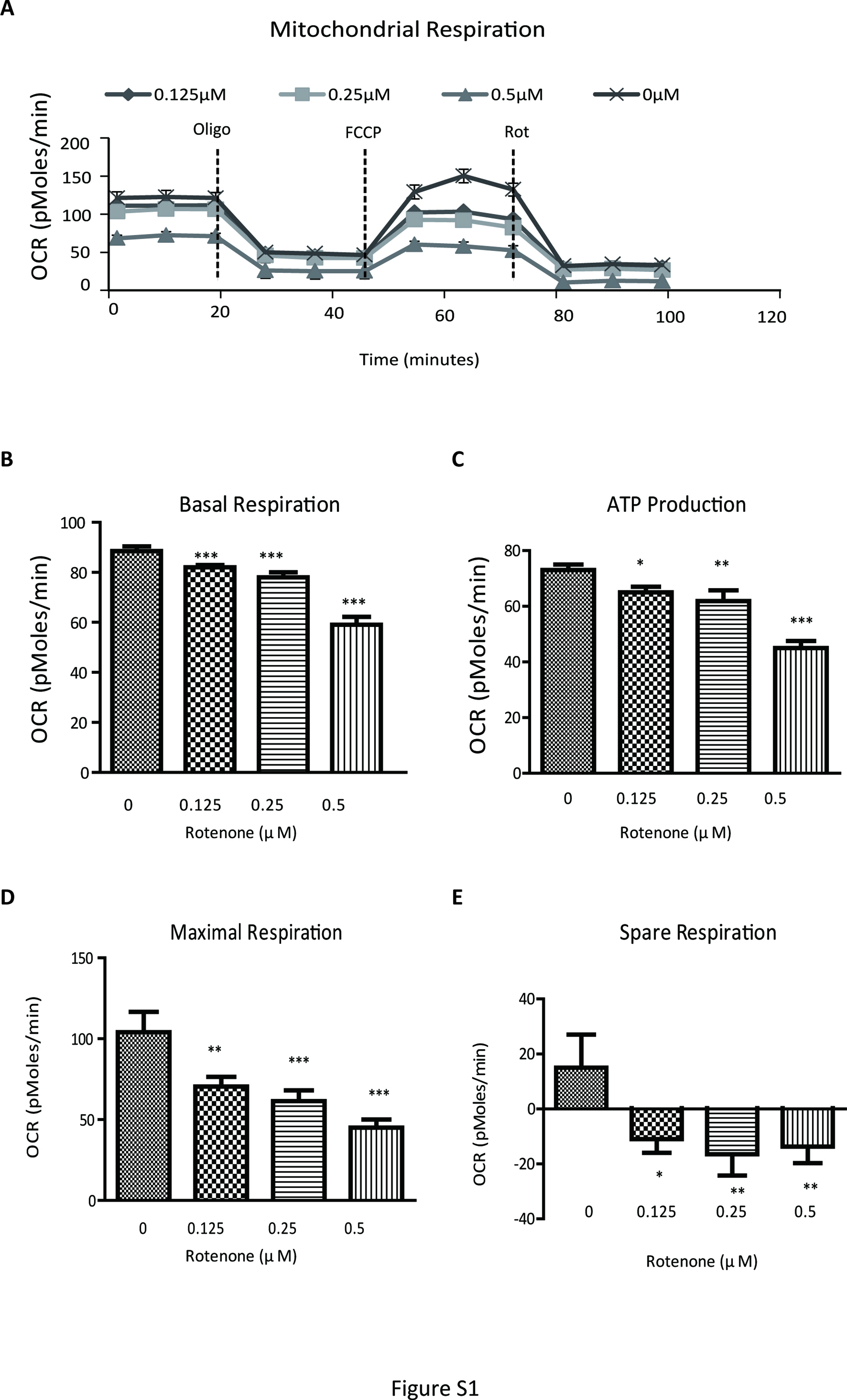

Supplement: Supplemental Figure 1 [file cddis2016358x2.tif]

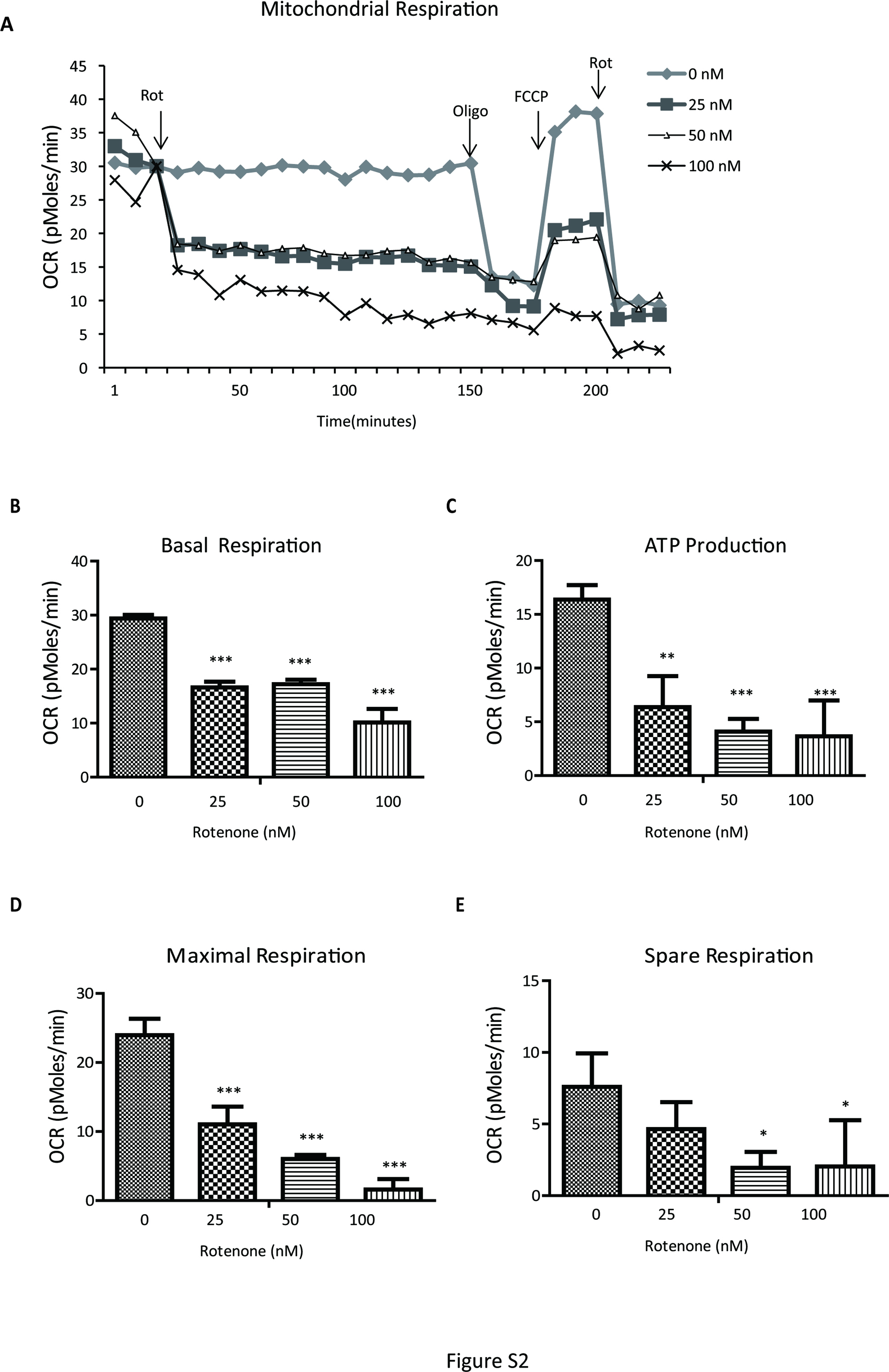

Supplement: Supplemental Figure 2 [file cddis2016358x3.tif]

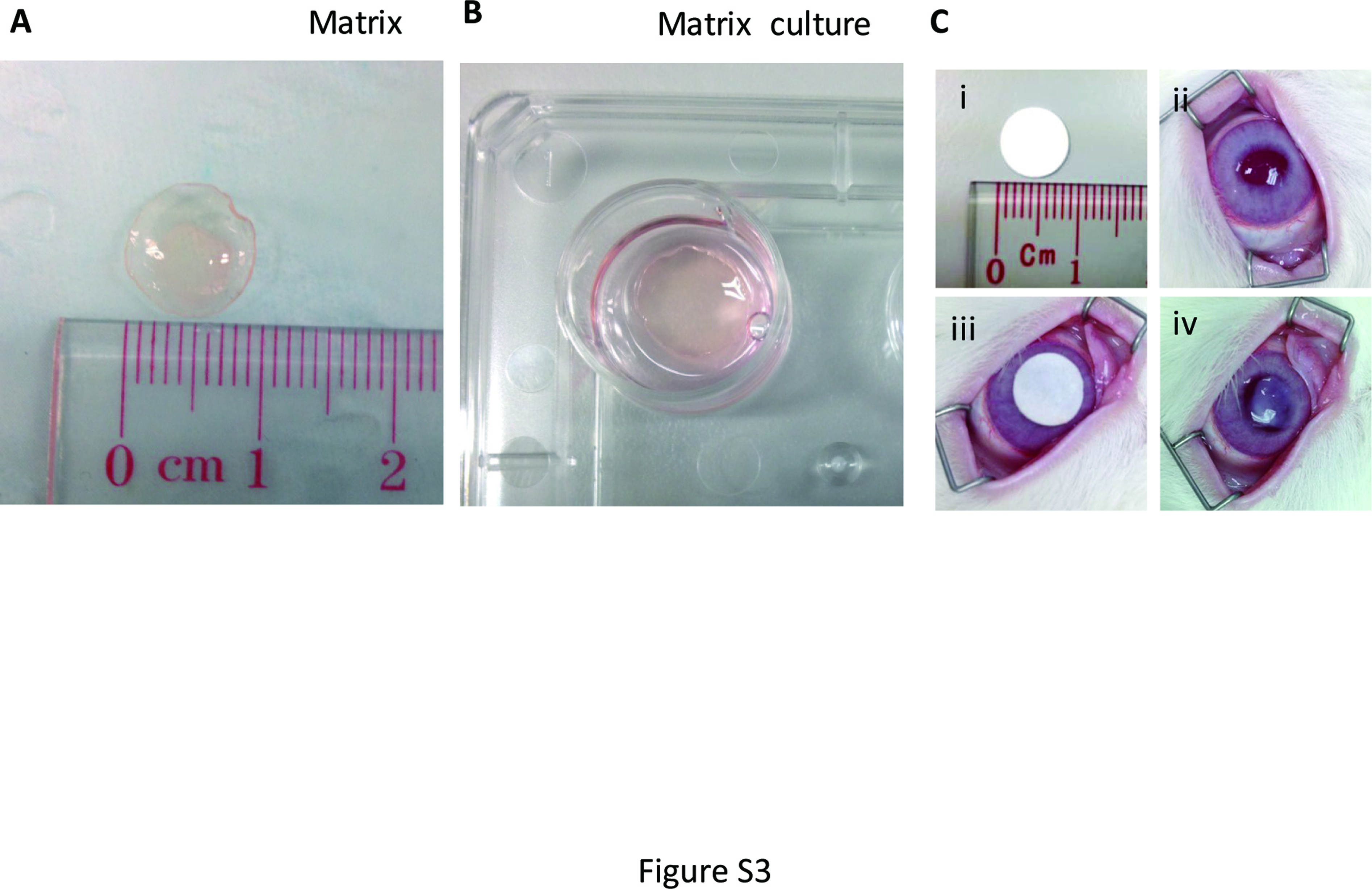

Supplement: Supplemental Figure 3 [file cddis2016358x4.tif]

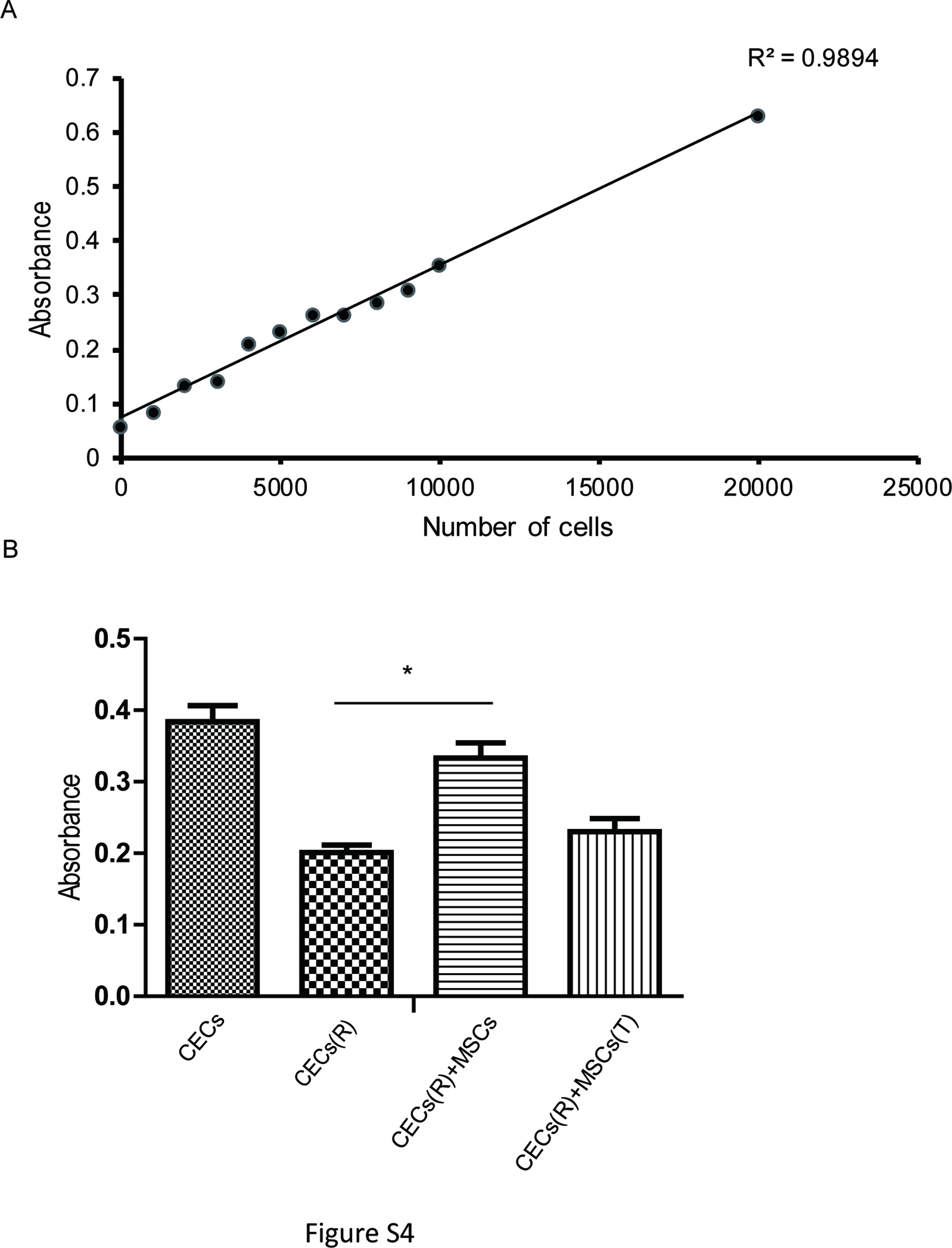

Supplement: Supplemental Figure 4 [file cddis2016358x5.tif]
